# Machine Learning Evaluation of Biliary Atresia Patients to Predict Long-Term Outcome after the Kasai Procedure

**DOI:** 10.3390/bioengineering8110152

**Published:** 2021-10-22

**Authors:** Martina Caruso, Carlo Ricciardi, Gregorio Delli Paoli, Fabiola Di Dato, Leandro Donisi, Valeria Romeo, Mario Petretta, Raffaele Iorio, Giuseppe Cesarelli, Arturo Brunetti, Simone Maurea

**Affiliations:** 1 Department of Advanced Biomedical Sciences, University of Naples “Federico II”, 80131 Naples, Italy; greg.dellipaoli@gmail.com (G.D.P.); lean.donisi@gmail.com (L.D.); valeria.romeo@unina.it (V.R.); brunetti@unina.it (A.B.); maurea@unina.it (S.M.); 2 Department of Electrical Engineering and Information Technology, University of Naples “Federico II”, 80125 Naples, Italy; carloricciardi93@gmail.com; 3 Bioengineering Unit, Institute of Care and Scientific Research Maugeri, 82037 Telese Terme, Italy; giuseppe.cesarelli@unina.it; 4 Department of Translational Medical Sciences, University of Naples “Federico II”, 80131 Naples, Italy; fabydd88@gmail.com (F.D.D.); petretta@unina.it (M.P.); raffaele.iorio@unina.it (R.I.); 5 Department of Chemical, Materials and Production Engineering, University of Naples “Federico II”, 80125 Naples, Italy

**Keywords:** artificial intelligence, bilirubin, ultrasound, magnetic resonance, shear-wave elastography

## Abstract

Kasai portoenterostomy (KP) represents the first-line treatment for biliary atresia (BA). The purpose was to compare the accuracy of quantitative parameters extracted from laboratory tests, US imaging, and MR imaging studies using machine learning (ML) algorithms to predict the long-term medical outcome in native liver survivor BA patients after KP. Twenty-four patients were evaluated according to clinical and laboratory data at initial evaluation (median follow-up = 9.7 years) after KP as having ideal (*n* = 15) or non-ideal (*n* = 9) medical outcomes. Patients were re-evaluated after an additional 4 years and classified in group 1 (*n* = 12) as stable and group 2 (*n* = 12) as non-stable in the disease course. Laboratory and quantitative imaging parameters were merged to test ML algorithms. Total and direct bilirubin (TB and DB), as laboratory parameters, and US stiffness, as an imaging parameter, were the only statistically significant parameters between the groups. The best algorithm in terms of accuracy, sensitivity, specificity, and AUCROC was naive Bayes algorithm, selecting only laboratory parameters (TB and DB). This preliminary ML analysis confirms the fundamental role of TB and DB values in predicting the long-term medical outcome for BA patients after KP, even though their values may be within the normal range. Physicians should be alert when TB and DB values change slightly.

## 1. Introduction

In the past decades, due to the growth of medical information digitalization and thanks to the availability of increasingly sophisticated technological quantitative tools, large volumes of patient data have become widely available. In this scenario, new approaches from computational sciences can be used to analyze medical data to extract critical health information that can help clinicians in the decision-making process and prognostic evaluation [1]. In particular, machine learning (ML) has gained great interest thanks to cheaper computing power and inexpensive memory and also because it is agnostic to the domain of application. It is a methodology of data analysis, a branch of artificial intelligence, that enables systems to learn and improve from data [2]. The ML methodology is spreading in clinical research with applications in several medical fields, such as neurology, cardiology, ophthalmology, pediatrics, and fetal monitoring [3,4,5,6,7,8,9,10,11].

Biliary atresia (BA) is a rare cholangiopathy of unknown etiology, which is characterized by inflammatory obliteration of both intrahepatic and extrahepatic bile ducts [12,13,14,15]; an early diagnosis is needed, and Kasai portoenterostomy (KP) represents the treatment of choice [16,17]. During the post-surgical follow-up, diagnostic evaluation consists of monitoring clinical and laboratory data as well as performing abdominal ultrasound (US) and magnetic resonance imaging (MR) [18,19,20,21,22]. In the literature, a good correlation of qualitative imaging findings using US and/or MR with the medical outcome of BA patients with native liver after KP during follow-up was described, as well as the potential role of US and MR findings in predicting the long-term medical outcome in such patients [21,22].

The aim of this study was to compare the accuracy of several quantitative parameters extracted from different methodologies, such as laboratory tests, US, and MR imaging, using ML algorithms in predicting the long-term medical outcome for native liver survivor patients with BA who have undergone KP.

## 2. Materials and Methods

### 2.1. Patient Population

Native liver survivor patients with BA after KP were retrospectively enrolled from the pediatric liver unit (January 2012 to December 2019). Exclusion criteria were (1) patients with liver transplantation and (2) patients with a time interval between acquisition of imaging studies (US and MR) greater than 30 days. Patients were initially evaluated by clinical, laboratory, and imaging (US and MR) studies to assess the medical outcome after KP. Patients were classified as having an ideal or a non-ideal medical outcome after KP following the criteria suggested by Ng et al. [23] and modified by Lee et al. [18]. An ideal medical outcome was defined as normal laboratory parameters with no evidence of medical complications of chronic liver disease (CLD), while a non-ideal medical outcome was based on at least one abnormal laboratory parameter and/or one CLD medical complication [18], including cholangitis, portal hypertension, variceal bleeding, fractures, hepatopulmonary syndrome, and portopulmonary hypertension. Successively, patients were similarly re-evaluated during long-term follow-up from initial evaluation to assess the disease course as stable or non-stable. The disease course was considered stable when the patient medical outcome remained unchanged at re-evaluation, whereas the disease course was considered non-stable when the patient medical outcome changed at re-evaluation to ideal from non-ideal or to non-ideal in progression; in particular, the status of non-ideal in progression consisted of the occurrence of at least one additional laboratory or clinical abnormality.

### 2.2. Laboratory Tests

The following laboratory parameters were used: white blood cell (WBC) count (n.v. > 4000/mm^3^), platelet (PLT) count (n.v. > 150,000/mm^3^), total bilirubin (TB; n.v. < 1.2 mg/dL), direct bilirubin (DB; n.v. < 0.5 mg/dL), albumin (n.v. > 3.5 g/dL), international normalized ratio (INR; n.v. < 1.3), alanine aminotransferase (ALT; n.v. < 40 IU/L), aspartate aminotransferase (AST; n.v. <40 IU/L), and γ-glutamyl transpeptidase (GGT; n.v. < 55 IU/L).

### 2.3. US and MR Imaging Acquisition and Processing

US and MR studies were acquired using imaging protocols, as previously reported [21].

For US quantitative analysis, the right hepatic lobe diameter and portal vein diameter were measured, as well as liver stiffness being analyzed using shear-wave elastography (SWE). In particular, the right hepatic lobe diameter (mm) was obtained on the midclavicular plane using the upper margin of the liver as the uppermost edge under the dome of the diaphragm, while the lower margin was taken as the lowermost edge of the lobe [24]; the portal vein was visualized in its longitudinal axis, and the greatest anteroposterior diameter at the liver hilum was measured in millimeters. SWE evaluates tissue stiffness, expressed as Young’s modulus (kPa) [25,26,27]. The position of the regions of interest (ROIs) is selected by the operator in real-time grayscale mode imaging, allowing them to choose a homogeneous vessel-free area placed at least 1 cm below the liver capsule [28]. For spleen diameter measurement (mm), the longitudinal dimension in the coronal plane was obtained; of note, the longitudinal measurement was performed between the most superomedial and the most inferolateral points [24].

**Figure 1:**
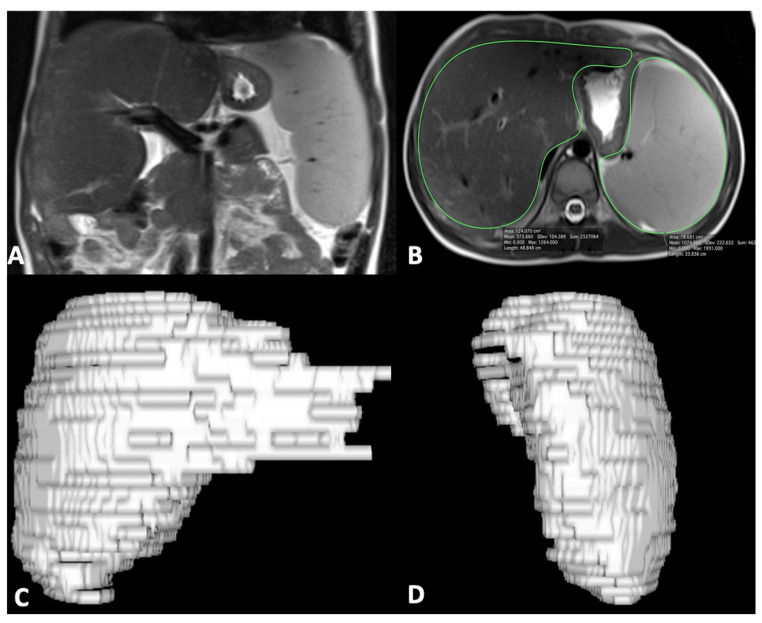
Coronal MR image shows liver and spleen enlargement (**A**); axial MR image shows ROI analysis of liver and spleen (**B**) to obtain 3D liver (**C**) and spleen (**D**) volume reconstruction images; of note, ROI analysis was performed on multiple sequential slices for completely including the liver and spleen.

For MR quantitative analysis, liver and spleen volumes were measured using a semi-automatic method with OsiriX^®^ version 3.3 software. An expert abdominal radiologist manually traced liver and spleen contours at different levels on T2-weighted images with the closed polygon selection tool under the ROI tool button; the Grow Region (2D/3D Segmentation) tool in the ROI dropdown menu made it possible to automatically outline the remaining boundaries. The automatic generated outlines were hand-adjusted with the closed polygon selection tool and the repulsor tool to optimize the ROIs. After selecting all of the ROIs within the series, OsiriX^®^ automatically calculated the volume by multiplying the surface and slice thickness and then adding up individual slice volumes. OsiriX^®^ also provided 3D images using the ROI volume tool (Figure 1) [29]. Furthermore, the portal vein diameter was measured in millimeters on the axial T2-weighted sequence at the liver hilum.

### 2.4. Statistical Analysis

A preliminary statistical analysis was performed analyzing the data of each methodology, both by the laboratory tests and imaging, for giving an input to ML algorithms. In the light of the small sample size, a non-parametric Mann–Whitney test was performed to distinguish stable (group 1) from non-stable (group 2) patients, considering each quantitative variable associated with the three diagnostic methodologies under examination, namely laboratory, US, and MR parameters. A Wilcoxon signed-rank test was performed to compare paired data. Moreover, a chi-square test was performed to compare the evaluation metrics (accuracy, sensitivity, specificity) of the different methodologies, since the augmentation of the data made the dataset not paired; the first two best evaluation metrics among laboratory tests, US, and MR parameters were compared. For all statistical tests, a two-tailed *p*-value of <0.05 was considered statistically significant. All the statistic tests were implemented using IBM SPSS Statistics (version 26).

### 2.5. Machine Learning: Tools and Algorithms

The data of all parameters were merged, and a selection method was used to understand the most important parameter among all diagnostic methodologies. Considering the small sample size and to make a fair comparison among the methodologies through machine learning analysis, an oversampling technique was performed, resulting in the generation of artificial data, namely the Synthetic Minority Oversampling Technique (SMOTE) proposed by Chawla et al., in order to double the amount of data [30,31]. This technique creates synthetic examples in the feature space from randomly selected pairs of real word–feature examples.

Because of the negative effect of irrelevant attributes on most ML schemes, it is common to precede learning with a feature selection stage that strives to eliminate all the redundant and irrelevant attributes for the classification and to identify the most informative features for the specific classification task. Dimensionality reduction yields a more compact and easily interpretable representation of the target concept, focusing the user’s attention on the most relevant variables. A wrapper method was used for feature selection before the final classification procedure when the features of all the methodologies were merged [32]. To classify the prognosis of the patients (stable or non-stable), different ML classification techniques were tested to ensure the best performance. As a result, classification methods, including supervised learning with a random forest (RF), naive Bayes (NB) algorithm, k-nearest neighbor (kNN) algorithm, and support vector machine (SVM), were evaluated [33,34]. In particular, an RF is composed of a large number of decision trees, which are mainly used to correct the overfitting problem of decision trees, which is surely an added value in this study with a small sample size. In this technique, multiple decision trees, trained from different subsets of the same training set, are averaged, and overfitting is avoided by reducing the variance of the system. The training algorithm works by applying bagging and randomization to tree learners. In this paper, the RF was made up of 100 models, used the information gain ratio as a split criterion, and had a tree depth of 10. Differently, the NB algorithm is a probabilistic ML algorithm based on Bayes’ theorem that calculates the probability of each class for a specified instance and then returns the class with the highest probability. This algorithm, requiring little data for training and little storage space, is suitable for the small size of the data sets at disposal. The kNN algorithm is an instance-based statistical method that works on the idea that the instances of a dataset are in proximity with other instances that have similar characteristics. In this classification approach, a test example is classified by observing the class label of its adjacent neighbors. The kNN algorithm finds out the k-nearest instances to the one to be classified and identifies its class on the basis of the most common class label. In this study, a *k* value was set equal to 3 and the Euclidean distance was used as the distance metric to identify the closest neighbors. Another instance-based algorithm is the SVM, which creates, in a binary classification, a hyperplane that separates data from two different classes. The largest possible distance is established between the separating hyperplane by maximizing the margin, thus creating the separation. The choice of kernel determines the separation boundary of the classes. The radial basis function (RBF) or Gaussian kernels are the most popular kernels used as default for any nonlinear model; polynomial kernels are also popular. An SVM with an RBF kernel was considered in this study.

The feature importance of the best subset of the features was computed according to the information gain for one of the best algorithms.

Leave-one-out cross-validation (LOOCV) was performed to evaluate the performance of the predictive models [35]. In LOOCV, every instance is in turn used to test the model induced from the other instances, ensuring the instance independence assumption, namely every prediction in LOOCV is independent of the other. This technique uses for each train/test round the biggest-possible train set, thus reducing the errors and being the most reliable validation method.

Standard evaluation metrics such as accuracy, sensitivity, and specificity, as well as the area under the curve of the receiver operating characteristic (AUCROC) were used to evaluate the models’ performance [36]. The AUCROC was computed by using as input a column with the real class and a second one with the probabilities that a record is classified as being from the selected class. The ML analysis was performed by means of the KNIME analytics platform (version 4.1.3) [10,37,38,39].

## 3. Results

### 3.1. Patient Population

The study population consisted of 24 patients (15 male; median age = 9.25 years, range = 5–25 years) according to inclusion and exclusion criteria. The median timing between the birth and KP surgical intervention was 67.5 days (range = 38–119 days). At initial evaluation, 15 patients had an ideal medical outcome, while the remaining 9 had a non-ideal medical outcome after KP. The median follow-up timing at initial evaluation after KP treatment was 9.7 years (range = 5–25 years) for all patients. At re-evaluation, after additional 4 years of long-term follow-up, 12 (50%) patients were stable (group 1) in their disease course, of which 9 had an ideal medical outcome and 3 a non-ideal medical outcome (Table 1); the other 12 (50%) patients had a non-stable (group 2) disease course, of which 6 patients changed from an ideal to a non-ideal medical outcome and 6 patients showed clinical disease progression (Table 2).

### 3.2. Descriptive Analysis

**Figure 2:**
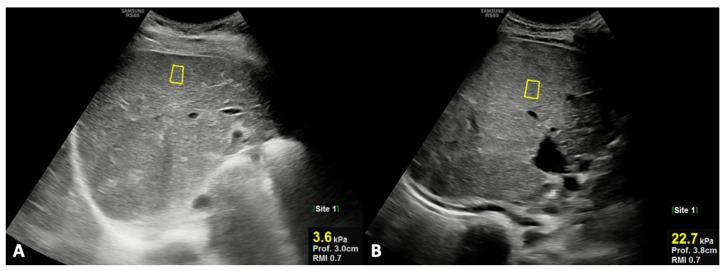
Ultrasound oblique scans under the right rib, showing shear-wave elastography measurements using ROI analysis in a patient of group 1 ((**A**) #5; Table 1—liver stiffness = 3.6 kPa) and in a patient of group 2 ((**B**) #11; Table 2—liver stiffness = 32.5 kPa).

The results of each diagnostic parameters, either by laboratory tests or imaging (US and MR), are reported in Table 3; in particular, TB and DB, as laboratory parameters, and US stiffness, as the imaging parameter, were the only statistically significant parameters between groups 1 and 2. In detail, TB and DB were significantly higher in patients of group 2 compared to those of group 1, even though the corresponding values in group 2 were still in the normal ranges. However, in patients of group 2, the mean values of TB (1.23 ± 0.43 vs. 0.74 ± 0.25; *p* = 0.005) and DB (0.53 ± 0.18 vs 0.29 ± 0.12; *p* = 0.006) were significantly increased at re-evaluation during the long-term follow-up; in particular, in the majority (75%) of patients of group 2, a significant increase in TB and DB values beyond the high normal limit was observed. Finally, US liver stiffness by SWE was significantly higher in patients of group 2 compared with those of group 1 (Figure 2).

### 3.3. Machine Learning

The result of SMOTE assessment increased the dataset from 24 to 48 subjects. Then, ML algorithms were implemented to classify the outcomes for all subjects using laboratory, US, and MR parameters by performing LOOCV (Table 4, Table 5 and Table 6). Table 3 contains the list of laboratory and imaging parameters that were given as input to the algorithms. For laboratory algorithms (Table 4), the RF was the best according to accuracy, sensitivity, and specificity values, even though the kNN algorithm achieved the highest AUCROC value. For US algorithms (Table 5), the RF was the best according to accuracy, sensitivity, and AUCROC, while NB and kNN algorithms obtained the highest specificity. For MR algorithms (Table 6), the kNN and SVM were the best according to accuracy, sensitivity, and specificity values, even though the kNN algorithm showed the highest AUCROC. The comparison of the mean performance between laboratory and imaging algorithms showed that the laboratory algorithms achieved the best results in terms of accuracy, sensitivity, and specificity values, as well as the AUCROC. The comparison between the first two best evaluation metrics (each best one is marked in bold for each methodology in Table 4, Table 5 and Table 6) among all the methodologies (laboratory, US, and MR) showed that the accuracy and sensitivity obtained through the RF applied on the laboratory data were greater than the others in a statistically significant way (*p*-value = 0.046 for both). When laboratory or imaging parameters were merged and analyzed as input to ML algorithms, using the wrapper technique as the feature selection method, the best algorithm was the NB algorithm using only laboratory parameters, such as TB and DB; however, the same result was obtained with the RF and kNN algorithms but using either laboratory or imaging parameters (Table 7). For the NB algorithm, the feature importance was also computed, thus determining that the TB contributed to the prediction with 56%, while DB contributed with 44%.

## 4. Discussion

In BA patients surviving with native liver after KP, the evaluation of the disease course and biliary cirrhosis occurrence is clinically relevant during follow-up [18,23]. For this purpose, clinical evaluation as well as laboratory tests and imaging studies are conventionally used. Imaging exams such as US and/or MR are able to depict liver and spleen anatomic conditions, providing a series of specific imaging parameters to assess the disease course [21]. Therefore, a wide spectrum of diagnostic parameters (clinical, laboratory, and imaging) is available in this setting, even though it is not well established how to use them and whether a complementary role may be hypothesized. In this study, the accuracy of several diagnostic quantitative parameters extracted from different methodologies, such as laboratory tests and imaging exams (US and MR), using ML algorithms was compared to predict the long-term medical outcome for native liver survivor patients with BA who have undergone KP. In detail, the patient population consisted of 24 patients, of which 50% were stable (group 1) in their disease course as an ideal (*n* = 9) or anon-ideal (*n* = 3) long-term medical outcome; conversely, the other 50% of the patients showed a long-term non-stable (group 2) disease course, since 6 patients changed from the ideal to the non-ideal medical status, while 6 patients had clinical disease progression. In this investigation, to predict the long-term medical outcome, laboratory parameters such as WBC and PLT counts, TB, DB, albumin, INR, ALT, AST, and GGT values were considered, as well as quantitative imaging parameters of liver and spleen conditions by US (right hepatic lobe diameter, portal vein diameter, and liver stiffness) and MR (liver and spleen volumes and portal vein diameter) imaging modalities. In this setting, laboratory data reflect mainly liver function, while imaging parameters are an expression of liver and spleen morphological changes, the liver parenchyma structure using the assessment of liver stiffness by US, and portal hypertension by measuring the portal vein diameter by both imaging techniques. ML algorithms with different operating principles were used to obtain a wider range of investigation. The overall results of the ML analysis showed that TB and DB as laboratory tests and US liver stiffness as the imaging parameter were the only significant parameters that were able to distinguish stable from non-stable patients in predicting the long-term medical outcome. These findings are reasonable since they reflect liver conditions, either directly in terms of the liver structure by US stiffness or indirectly by TB and DB reflecting liver function. These observations are concordant and confirm previous experiences in which a predictive role of serum bilirubin levels and US liver stiffness has been suggested in patients with BA treated with KP during early and long-term follow-up [19,40,41,42,43,44]. In particular, among the used ML algorithms, the RF algorithm was the best either for laboratory or for US parameters, while SVM and kNN algorithms were the best according to MR parameters. However, the evaluation of the mean performance of laboratory and imaging algorithms showed that laboratory algorithms achieved the best results in terms of accuracy, sensitivity, and specificity values, as well as the AUCROC. Furthermore, when all the diagnostic parameters, either by laboratory tests or by imaging, were merged and analyzed as input to ML algorithms, the best algorithm was the NB algorithm using only TB and DB, even though the same result was obtained also with RF and kNN algorithms using either laboratory or imaging parameters. Of course, the high evaluation metrics achieved could make a reader think of overfitting, since a high number of computations on a small sample of data through simple cross-validation provide an optimistic estimation of the model, as reported by Tsamardinos et al. [45]. However, it is worth underlining that the best results were obtained by using the combination of LOOCV and the RF, both of which are used to reduce the chance of overfitting. Moreover, it should be emphasized that the purpose of the article was not to obtain a perfect model, since the dataset had obtained an injection of artificial data, but to understand the weight and importance of the parameters extracted from US, MRI, and laboratory tests in predicting the long-term outcome for native liver survivor patients with BA after KP. Indeed, ML has already been used to compare different clinical methodologies to predict an outcome (both diagnostic and prognostic) in cardiology or choose the best resolution for ultrasound [9,46,47].

Thus, this preliminary ML evaluation confirms that laboratory tests, specifically TB and DB, represent powerful parameters to predict the long-term medical outcome in native liver survivor patients with BA after KP, supporting previous observations that already suggested a main role of serum bilirubin levels for this purpose [19]. These preliminary results and those of previous investigations may have significant advantages in terms of clinical patient management and cost-effectiveness, since TB and DB plasma measurements as laboratory tests are easily performed, widely available, and not expensive [19,42]. However, even though the values of TB and DB were able to predict the long-term medical outcome, they were still in the normal range but tended toward the upper limit; of note, this trend was confirmed by increased values of TB and DB beyond the high normal limit at re-evaluation in the majority of patients with non-stable disease.

To date, ML methods have been applied in clinical research with applications in several medical fields, of which many are in pediatric diagnostic imaging [48,49,50]. In particular, the ML methodology has been applied to assess skeletal maturity on hand X-rays [51], to diagnose and classify acute appendicitis using laboratory tests and US [52], to identify MR biomarkers of the autistic spectrum [53], and to evaluate CLD using clinical data and MR [54]. Furthermore, recent studies have suggested a role of ML methodologies also in patients with BA, focusing on disease diagnosis. In detail, Hoshino et al., using an ML algorithm, realized an iPhone application (Baby-Poop) able to capture subtle differences in stool color that may be undetectable by a layperson to get early diagnosis of BA [55]. A similar ML application with the same purpose was made by Angelico et al., who created PopòApp [56]. Moreover, Zhou et al. developed an ensembled deep learning model to facilitate the diagnosis of BA for non-expert radiologists using DB values and US images as well as videos of the gallbladder [57]. In this setting, this pilot experience is the first that reports an ML evaluation using laboratory and imaging parameters with long-term predictive purposes in patients with BA after KP, supporting the main role of laboratory tests in the follow-up of such patients. A future development could be the use of deep learning algorithms on the images to further test their feasibility to predict the outcome.

Some limitations of this study should be addressed. Mainly, the small sample size and the retrospective type of the investigation might be not optimal, but the low incidence of BA, a rare pediatric disease, should be considered; therefore, additional experiences in a larger patient population are required. The data used in ML analysis to establish the long-term medical outcome consisted of laboratory and quantitative imaging parameters as continuous variables requested by ML algorithms; therefore, the presence or absence of CLD medical complications was not included in the analysis for the lack of continuous quantification; similarly, patients with asplenia or poly-splenia, possible findings in children with BA, may be not included. Moreover, technical ML limitations were also present, particularly due to the implementation of SMOTE for augmenting data; nevertheless, predicting the outcome was not the main purpose of the research, since the aim was to compare the accuracy of several diagnostic parameters extracted from different methodologies. Therefore, the use of SMOTE, which was used to augment the dataset with artificial data, as already done in a previous study, rather than to balance a minority class, as is usually employed [58], might have a limited impact on the analysis; in comparison with traditional logistic regression, ML has the advantage of not requiring the assessment of assumptions to be performed, such as the detection of outliers or a strict limit between subjects and variables. Moreover, ML algorithms have demonstrated empirically their powerfulness in several fields. The main disadvantage of ML algorithms is the black-box style, since the input and output of the algorithms are known but a numerical model is not provided; nevertheless, ML algorithms may be used as clinical support decision-making systems since they provide users with a probability for each subject of being part of a fixed class.

In conclusion, the results of this preliminary ML investigational study of native liver survivor patients with BA who have undergone KP, integrating laboratory and imaging quantitative diagnostic data, showed that TB and DB represent the fundamental parameters to predict the long-term medical outcome after treatment, confirming the results of previous studies that demonstrated a main predictive role of serum bilirubin levels in such patients during early follow-up. In particular, the values of TB and DB may be within the normal range but with a slight increase; therefore, clinicians should be alert when the values of these laboratory parameters show subtle changes. Furthermore, US liver stiffness, reflecting liver parenchyma changes, is the best imaging parameter for this purpose.

## Figures and Tables

**Table 1:** Clinical results of stable patients (group 1).

#	Sex	Age (years)	Medical Status *	Laboratory Abnormalities °	CLD Complications
1	M	6	Ideal	-	-
2	M	13	Ideal	-	-
3	F	10	Ideal	-	-
4	M	9	Ideal	-	-
5	F	13	Ideal	-	-
6	M	6	Ideal	-	-
7	M	5	Ideal	-	-
8	F	9	Ideal	-	-
9	M	14	Ideal	-	-
10	M	11	Non-ideal	AST, ALT, WBC, PLT	Portal hypertension, cholangitis
11	M	9	Non-ideal	AST, ALT, GGT, WBC, PLT	Portal hypertension
12	M	25	Non-ideal	TB, PLT	Portal hypertension

* The medical status was established according to the criteria of Ng et al. [23] and Lee et al. [18]. ° Abnormal values out of the normal range. - = not present.

**Table 2:** Clinical results of non-stable patients (group 2).

#	Sex	Age (years)	Medical Status at Initial Evaluation *	Laboratory Abnormalities at Re-Evaluation	CLD Complications at Re-Evaluation	Long-Term Medical Outcome
1	M	13	Ideal	TB	-	Non-ideal
2	M	10	Ideal	TB	-	Non-ideal
3	M	12	Ideal	TB	Cholangitis	Non-ideal
4	M	5	Ideal	ALT, PLT	-	Non-ideal
5	F	14	Ideal	TB	Cholangitis	Non-ideal
6	F	6	Ideal	WBC	-	Non-ideal
7	M	6	Non-ideal ^a^	TB, PLT	Portal hypertension	Clinical progression
8	M	5	Non-ideal ^b^	WBC	-	Clinical progression
9	F	7	Non-ideal ^c^	AST, ALT, WBC,	-	Clinical progression
10	F	10	Non-ideal ^d^	TB	-	Clinical progression
11	M	7	Non-ideal ^e^	WBC	-	Clinical progression
12	F	7	Non-ideal ^f^	TB	-	Clinical progression

* The medical status was established according to the criteria of Ng et al. [23] and Lee et al. [18]. ° Abnormal values out of the normal range. - = not present. ^a^ Increased values of AST and ALT associated with the presence of cholangitis; ^b^ decreased values of PLT associated with the presence of portal hypertension; ^c^ decreased values of PLT associated with the presence of cholangitis and portal hypertension; ^d^ abnormal values of PLT and INR associated with the presence of cholangitis, portal hypertension, and variceal bleeding; ^e^ abnormal values of AST, ALT, GGT, and PLT associated with the presence of portal hypertension; ^f^ abnormal values of AST, ALT, INR, albumin, WBC, and PLT associated with the presence of cholangitis and portal hypertension.

**Table 3:** Laboratory and imaging results in group 1 and group 2.

-	Parameter	Group 1 (Mean ± SD)	Group 2 (Mean ± SD)	*p-*Value
Laboratory	AST (IU/L)	31 ± 11	40 ± 25	0.443
	ALT (IU/L)	29 ± 21	33 ± 20	0.291
	GGT (IU/L)	23 ± 19	25 ± 22	0.887
	**TB (mg/dL)**	0.38 ± 0.34	0.74 ± 0.25	**0.001**
	**DB (mg/dL)**	0.13 ± 0.09	0.29 ± 0.12	**0.001**
	INR	1.06 ± 0.07	1.12 ± 0.11	0.198
	Albumin (g/dL)	4.74 ± 0.24	4.44 ± 0.50	0.114
	WBC (cells/mm^3^)	6567 ± 2293	6122 ± 1873	0.551
	PLT (cells/mm^3^)	242083 ± 115800	188667 ± 93292	0.378
US	Portal vein (mm)	9.75 ± 1.60	9.08 ± 2.11	0.932
	Liver diameter (mm)	129.17 ± 23.53	114.00 ± 21.56	0.078
	Spleen diameter (mm)	118.00 ± 23.83	124.92 ± 25.65	0.443
	**Liver stiffness (kPa)**	5.95 ± 1.28	10.47 ± 7.32	**0.020**
MR	Portal vein (mm)	9.92 ± 1.38	8.75 ± 2.05	0.198
	Liver volume (cm^3^)	923.46 ± 250.47	823.97 ± 282.75	0.242
	Spleen volume (cm^3^)	300.64 ± 199.82	356.17 ± 142.86	0.198

Note: the parameters statistically significant are marked in bold.

**Table 4:** Results using laboratory features after the SMOTE technique in predicting long-term medical outcomes.

Algorithms	Accuracy (%)	Sensitivity (%)	Specificity (%)	AUCROC
**RF**	**95.8**	**95.8**	**95.8**	0.991
NB	72.9	62.5	83.3	0.866
kNN	93.8	91.7	**95.8**	**0.997**
SVM	89.6	87.5	91.7	0.896
Mean performance	88.0	84.4	91.7	0.937

Note: the best value for each evaluation metric is marked in bold.

**Table 5:** Results using US imaging features after the SMOTE technique in predicting long-term medical outcomes.

Algorithms	Accuracy (%)	Sensitivity (%)	Specificity (%)	AUCROC
RF	**79.2**	**79.2**	79.2	**0.868**
NB	64.6	41.7	**87.5**	0.642
kNN	**79.2**	70.8	**87.5**	0.818
SVM	75.0	70.8	79.2	0.750
Mean performance	74.5	65.6	83.4	0.769

Note: the best value for each evaluation metric is marked in bold.

**Table 6:** Results using MR imaging features after the SMOTE technique in predicting long-term medical outcomes.

Algorithms	Accuracy (%)	Sensitivity (%)	Specificity (%)	AUCROC
RF	79.2	79.2	79.2	0.878
NB	60.4	41.7	79.2	0.677
kNN	**83.3**	**83.3**	**83.3**	**0.908**
SVM	**83.3**	**83.3**	**83.3**	0.833
Mean performance	76.6	71.9	81.3	0.824

Note: the best value for each evaluation metric is marked in bold.

**Table 7:** Results using merged laboratory and imaging features after the SMOTE technique in predicting long-term medical outcomes.

Algorithms	Accuracy	Sensitivity	Specificity	AUCROC	Features Selected
RF	100	100	100	1	TB, US liver diameter, MR portal vein diameter
NB	100	100	100	1	TB, DB
kNN	100	100	100	1	TB, DB, WBC, US Stiffness, MR portal vein diameter
SVM	93.3	100	87.5	0.938	TB, INR

## Data Availability

Data are not available due to privacy policy.
